# Astaxanthin Is Able to Prevent Alcohol-Induced Dysfunction of Liver Mitochondria

**DOI:** 10.3390/antiox11102019

**Published:** 2022-10-12

**Authors:** Olga Krestinina, Irina Odinokova, Linda Sotnikova, Roman Krestinin, Alena Zvyagina, Yulia Baburina

**Affiliations:** Institute of Theoretical and Experimental Biophysics, Russian Academy of Sciences, Pushchino 142290, Moscow Region, Russia

**Keywords:** rat liver mitochondria (RLM), chronic ethanol intoxication, the activity of respiratory chain complexes, astaxanthin (AX)

## Abstract

The search for new targets for the pathological action of ethanol remains an urgent task of biomedicine. Since degenerative changes in the liver are associated with the development of oxidative stress, antioxidants are promising agents for the treatment of alcohol-related diseases. In this work, we studied the ability of the carotenoid antioxidant, astaxanthin (AX), to prevent ethanol-induced changes in the liver of rats. It was shown that AX is able to protect the structure of mitochondria from degenerative changes caused by ethanol to improve mitochondrial functions. AX positively influences the activity and expression of proteins of the mitochondrial respiratory chain complexes and ATPase. In addition, a protective effect of AX on the rate and activity of mitochondrial respiration was demonstrated in the work. Thus, studies have shown that AX is involved in protective mechanisms in response to ethanol-induced mitochondrial dysfunction.

## 1. Introduction

The problem in the treatment of diseases caused by chronic alcohol abuse is still relevant throughout the world. It is known that the destructive effect of alcohol affects all organs and tissues and is the cause of many diseases. Alcohol primarily affects the liver, causing various damages.

Under the influence of alcohol, redox processes are disrupted, hepatocytes are damaged, and the toxic breakdown product of alcohol, acetaldehyde, accumulates [1]. The liver increases in size and its function is impaired. As a result, inflammation develops in the tissue and the liver cells are replaced by connective tissue. Synthesis of connective tissue leads to fibrosis, which in turn ends in cirrhosis of the liver, which is considered the main reason for the transplantation of this organ. There is a lot of evidence that pathological changes in liver, which are the result of chronic alcohol intoxication, are associated with oxidative stress [2,3]. Under normal conditions, the balance between ROS and antioxidants is maintained in mitochondria. However, under pathological conditions, an excess of free radicals can lead to DNA damage, degradation of proteins and, as a result, to numerous diseases [4]. It is known that in mitochondria, oxidative stress leads to disruption of the functioning of the respiratory chain complexes, which results in dysfunction of the oxidative phosphorylation system (OXPHOS), and consequently, to a decrease in ATP production [4]. The mitochondrial respiratory chain complexes consist of five complexes: complexes I, II, III, IV, and V. Their functions include the catalysis of ADP to ATP [5] and the operation of the electron transport chain and other important pathways in mitochondria [6]. Under the influence of pathological factors (including alcohol), there are violations of the structure and functioning of these complexes, as well as the OXPHOS system as a whole [7,8].

In this regard, numerous antioxidants of various nature are widely studied with the aim of reducing the pathological effects of alcohol [9,10]. It has been shown that the antioxidant properties of benzodiazepines help to overcome the pathological consequences of alcohol withdrawal syndrome [11]. The polyphenols contained in olive oil also help in the dealing with the consequences of alcohol intoxication due to their antioxidant properties [12]. Recently, studies have appeared devoted to the study of the protective effect of astaxanthin (AX) on alcohol-induced pathological changes in various organs and tissues [13,14,15,16]. AX is a strong natural antioxidant of the carotenoid group, found in algae, shrimp, fish, and other marine organisms [17]. Since it is not synthesized in the human body, it can only be obtained from food. In addition to antioxidant properties, its spectrum of action also includes anti-inflammatory [16,17], anti-aging, and cardioprotective properties [18,19]. Despite numerous evidence on the protective effect of AX on liver cells and tissues during alcohol intoxication, the mechanism of this action remains the subject of debate [14,15,16].

In our study, it was shown that AX has a protective effect on the rat heart mitochondria in conditions of heart failure [20,21,22]. There was an improvement in mitochondrial functions, an increase in the activity of respiratory chain complexes, and inhibition of the opening of a mitochondrial non-specific pore (mitochondrial permeability transition pore, mPTP). We suggest that AX is directly involved in the mechanisms of the protective action of mitochondria in response to pathological conditions. In this investigation, we investigated whether AX exhibits protective properties in rat liver mitochondria during chronic alcohol intoxication.

## 2. Materials and Methods

### 2.1. Animals and Treatment

Four groups of animals were used in the experiments: group 1 (control), group 2 (treated with AX (Natural, China)), group 3 (treated with ethanol), and group 4 (treated with ethanol and AX together). At the start of the experiment, the average weight of the rats was 167.87; during the experiment, the weight gain was 163.12 ± 11.55 g. In our experiments, we used the Lieber–DeCarly model of chronic alcohol intoxication (the rats of groups 3 and 4), which allows one to achieve the consumption of alcohol at high doses [23]. Mixtures for preparing liquid nutrition were manufactured by BioServ (Frenchtown, NJ, USA). The control diet contained fats, proteins, carbohydrates, trace elements, and vitamins, with 18% of the total calories coming from proteins, 35% from fats, and 47% from carbohydrates. In the alcohol diet, 36% of the calories from the carbohydrate components were replaced by calories from ethanol, the concentration of which was 5% in the final diet. Rats that received an alcoholic diet had free access to food throughout the day, and the control rats received an amount of food equivalent to that consumed by their paired alcoholic rats; food consumption was measured daily. Over a 10 day period of habituation, the rats received a gradually increasing amount of ethanol (0, 1, 2, 3, 4, and 5%) in their food, and then all alcoholic rats received food containing 5% ethanol for 8 weeks. The rats consumed an average of 60–80 calories daily, and alcoholic rats received 14.86 ± 16.25 g of ethanol per 1 kg rat weight, which is consistent with the published data. The rats of group 3 and 4 were orally administered a weighed quantity of 5% AST (150 mg/kg) dissolved in olive oil. The animals of the groups 1 and 3 received an equal amount of olive oil as a vehicle. AX (5%, 150 mg/kg) was administrated as described in [22,24]. The experiments were carried out according to the Regulations for Studies with Experimental Animals (Decree of the Russian Ministry of Health of 12 August 1997, No. 755). The protocol was approved by the Commission on Biological Safety and Ethics at the Institute of Theoretical and Experimental Biophysics, Russian Academy of Sciences (February 2021, protocol N18/2021).

### 2.2. Histological Analysis

For histological analysis, fragments of the periportal zone of the liver lobules were quickly cut with a scalpel from the whole liver immediately after removal from the abdominal cavity and quickly washed with cold phosphate-buffered saline to remove blood. Then the samples were fixed in neutral buffered formalin for 24 h at room temperature according to the standard method. After fixation was completed, the fragments were washed three times to remove excess phosphates in distilled water and immersed in O.K.T. Compound Tissue Tek (Sakura, Tokyo, Japan) for 12 h at +4 °C. Sets of three consecutive 9 µm thick transverse sections were prepared using a Shandon CRYOTOME 620E (Thermo Fisher Sci., Waltham, MA, USA) in 30 µm increments. Each set of three adjacent sections was stained with hematoxylin and eosin (H&E) and two differential trichrome staining methods. Histotopograms were taken on a Nikon Eclipse Ti-E microscope station (Nikon, Tokyo, Japan) using Nis Elements AR4.13.05 software (Build933) to obtain a general picture of alcoholic liver damage.

Fibrosis as an indicator of alcoholic liver damage was assessed by two methods: Malory’s trichrome stain and Lilly’s trichrome stain. The percentage of fibrotic changes in the periportal zone of the liver was assessed from digitized images (at least five areas of analysis from each slice) using the non-commercial ImageJ software (https://imagej.nih.gov/ij/) (accessed on 18 May 2022). The degree of fibrosis was assessed by the increase in deposited collagen (blue areas on the section) in the tissue and was calculated as the area occupied by collagen expressed as a percentage of the total area of the analyzed area. Data are presented as mean ± standard deviation.

### 2.3. Isolation of Rat Liver Mitochondria

Rat liver mitochondria (RLM) were isolated from rats by the standard method using a homogenization medium containing 210 mM mannitol, 70 mM sucrose, 1 mM EGTA, 0.05% bovine serum albumin fraction V, and 10 mM Tris (pH 7.3). The homogenate was centrifuged at 800× *g* for 10 min to pellet the nuclei and damaged cells. The supernatant containing the mitochondria was centrifuged for 10 min at 9000× *g*. Sedimented mitochondria were washed twice in a medium without EGTA and BSA for 10 min at 9000× *g* and resuspended in the same medium. The protein concentration was then determined using a Bradford assay.

### 2.4. Evaluation of Mitochondrial Respiratory Functions

Mitochondria (1 mg protein/mL) were incubated at 25 °C in a medium containing 125 mM KCl, 10 mM Tris (pH 7.4), and 2 mM K_2_HPO_4_. In the experiments, glutamate (5 mM) and malate (5 mM) were used as respiratory substrates. The oxygen consumption rates (V_st.2_, V_st.3_, and V_st.4_; ng-atom O min−1 mg−1 of protein) were evaluated using the Record program (Pushchino, Russia). The change in the rate of oxygen consumption in different states, the rate of state 2 (V_st.2_), the rate of state 3 (V_st.3_), the rate of state 4 (V_st.4_), and the rate of uncoupled respiration (V_u_) were calculated as the change in the rate of oxygen consumption per minute per milligram of protein. The respiratory control index (RCI) was measured in a closed chamber after the addition of 150 μM ADP to RLM and was calculated as the ratio of V_st.3_ to V_st.4_. The phosphate to oxygen (P/O) ratio was calculated as the ratio of the amount of added ADP to the amount of O_2_ needed to convert ADP to ATP

### 2.5. Blue Native Electrophoresis (BNE) and Measurement of the Activity of Electron Transport Chain Complexes and ATP Synthase

The buffer containing 0.75 M aminocapronic acid, 50 mM Bis-Tris/HCl, pH 7, and 10% dodecyl maltoside was added to intact RLM, and the suspension was kept on ice for 20 min. After 10 min of centrifugation at 10,000× g, the supernatant was supplemented with 5% Serva Blue G dissolved in 1 M aminocapronic acid. Samples were applied onto 3%–13% gradient gel, 70 µg of the sample per lane. An HMW Calibration Kit for Native Electrophoresis (Sigma-Aldrich, St. Louis, MO, USA) was used as a molecular marker.

To preserve the activity of the complexes, electrophoresis was carried out at 4 °C. After the BNE, the in-gel activity of complexes I, II, IV, and V was determined as described [25]. The activity of the complex III was measured according to [26]. Mitochondrial samples were separated by one-dimensional BNE (1D BNE), and the gel was stained for ~10–30 min for the detection of activity of CI by buffer containing 100 mM Tris-HCl, pH 7.4, 0.14 mM NADH, and 1 mg/mL NBT (nitro blue tetrazolium chloride). For the detection of complex IV (CIV) activity, the gel was stained for 1 h by a buffer containing 10 mM KH_2_PO_4_ (pH 7.4), 1 mg/mL (3,3′-diaminobenzidine) (DAB), and 0.2 mg of cytochrome *c*. The complex II (CII) activity was determined by staining with a buffer containing 50 mM KH_2_PO_4_ (pH 7.4), 84 mM succinate, 0.2 mM phenazine methosulfate (PMS), and 2 mg/mL NBT. For the detection of CV activity, the gel was stained for 16 h with a buffer containing 10 mM ATP, 35 mM Tris (HCL), 270 mM glycine, 14 mM MgSO_4_, and 0.2% Pb(NO_3_)_2_. Further, the gels were placed in 10% acetic acid (to stop the reactions), washed with water, and scanned. Two BNEs were run in parallel, one gel was stained to determine the in-gel activity, and the other was stained for the excision of complexes and subsequent immunoblotting. Bands of separated complexes were excised after electrophoresis and applied in 12.5% SDS-PAGE followed by immunoblotting [27]. Precision Plus Pre-stained Standards markers from Bio-Rad Laboratories (Hercules, CA, USA) were used.

### 2.6. Electrophoresis and Immunoblotting of the Mitochondrial Proteins

Tissue lysate samples, ~ 6–7 mg of heart tissue from left ventricular, were prepared by adding ice-cold buffer (RIPA buffer and protease cocktail) to a piece of liver tissue in the suspension. After homogenization, the samples obtained were centrifuged at 10,000× *g* for 20 min. The resulting supernatants were solubilized in Laemmli buffer (Bio-Rad, Hercules, CA, USA) and applied to PAAG (12.5%) with following Western blot analysis. The amount of protein was 20 µg per lane. The polyclonal anti-ALT antibody (1:500), anti-AST (subunit *b*) (1:3000), and anti-LDG antibody (1:5000) were from Abcam (Cambridge, UK). The monoclonal anti-GAPDH antibody (Cell Signaling, Danvers, MA, USA) was used as a loading control. Immunoreactivity was detected using an appropriate secondary antibody conjugated to horseradish peroxidase (Jackson Immuno Research, West Grove, PA, USA). The blot was detected with ECL (Bio-Rad, Hercules, CA, USA) using the ChemiDoc Touch Imaging System (Bio-Rad, Hercules, CA, USA). Protein bands were quantified by densitometry (Image Lab program, Hercules, CA, USA).

Mitochondrial samples for determining the protein levels were prepared by placing the aliquots of isolated intact RLM in Eppendorf tubes and solubilizing them in Laemmli buffer (Bio-Rad, Hercules, CA, USA). After heating for 3 min to 95 °C, the samples containing 20 μg of mitochondrial protein were applied onto each line and subjected to electrophoresis. The monoclonal anti-ATPG1/G2/G3 antibody (subunit *c*) (1:1000), anti-ATPF1 (subunit *b*)(1:500), the monoclonal COX IV antibody (1:2000), and the Polyclonal TSPO antibody (1:1000) were from Abcam (Cambridge, UK); the monoclonal anti-CNPase antibody (1:10000) was obtained as described [28]. Alterations in the level of subunits of ETC complexes were determined using a Total Oxphos Rodent WB Antibody Cocktail (Abcam, Cambridge, UK). The Oxphos Antibody Cocktail consists of complex V alpha subunit (CV-ATP5A-55 kDa), complex III core protein 2 (Cytochrome b-c1 complex subunit 2, CIII-UQCRC2-48 kDa), complex IV subunit I (mitochondrially encoded cytochrome c oxidase I, CIV-MTCO1-40 kDa), complex II subunit 30 (Succinate dehydrogenase [ubiquinone] iron-sulfur subunit, CII-SDHB-30 kDa), and complex I subunit NDUF8 (NADH dehydrogenase (ubiquinone) 1 beta subcomplex subunit 8, CI-NDUFB8-20 kDa). The polyclonal antibody Tom20 (Santa-Cruz, Dallas, TX, USA) was used as a loading control.

### 2.7. Statistical Analysis

For statistical analysis, relative levels of protein density were expressed as means ± SDs from at least three independent experiments. The statistical significance of the differences between the pairs of mean values was evaluated using the Student–Newman–Keul test. The difference was considered significant at *p* < 0.05.

## 3. Results

In our experiments, we used the Lieber–DeCarly model of chronic alcohol intoxication, which allows high-dose alcohol intake [23]. Four groups of animals were used in the experiments: group 1 (control), group 2 (treated with AX), group 3 (treated with ethanol), and group 4 (treated with ethanol and AX together). Comparisons of the parameter body and liver weight of the animal, as well as blood alcohol content parameters, are presented in Table 1. At the beginning of the experiment, all animals were approximately the same weight (165 ± 2 g). As can be seen from the table, rats from the group 3 (received alcohol) had less weight than rats from other groups, while the liver of rats from the group 3 weighed more on average. At the same time, these parameters in rats of the group 4 (together with ethanol received AX) were comparable to the control ones. It should also be noted that the average blood alcohol content of rats of groups 3 and 4 was 0.9 g/l, which corresponds to average intoxication. The presented data allowed us to suggest alcohol-dependent degenerative changes in the liver of experimental animals.

**Figure 1:**
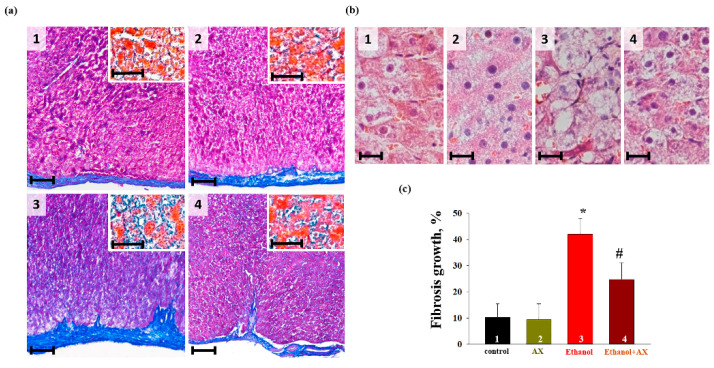
Histological samples of liver tissue in rats of the control and experimental groups. (**a**)—Fragments of histotopograms of periportal areas of rat liver tissue. Light microscopy; main images—Masson’s trichrome stain (collagen/fibrosis—blue, non-collagen components—pink, cell nuclei—dark crimson), ruler 100 µm; Inserts—Malory trichrome stain (collagen/fibrosis—blue, non-collagen components—orange/red, cell nuclei—red), ruler 50 µm; (**b**)—Photomicrographs of perivascular rat liver hepatocytes. Light microscopy; H&E (cell nuclei in purple, erythrocytes in red, cell cytoplasm in pink), ruler 20 µm; (**c**) is a diagram showing the growth of deposited collagen in the periportal region of the liver of each group of rats. The data are presented as the means ± SD of three independent experiments. * *p* < 0.05 significant difference in the protein levels in comparison with the control (group 1). # *p* < 0.05 compared to RLM isolated from ethanol-administrated rats (group 3). The statistical significance of the differences between the pairs of mean values was evaluated using the Student–Newman–Keul test.

At the next stage, we performed a histochemical analysis of liver tissue samples from each group of rats. To assess the degree and character of alcoholic damage and determine its localization, an analysis of the histotopograms of samples of the periportal zones of the liver tissue of all groups of animals was performed. In addition, the deposition of collagen in the walls of the central vessels (Figure 1a, main figures) and in the parenchyma (Figure 1a, insets) was assessed and compared. The comparison of the data obtained for the groups 1 and 2 did not reveal differences in any of the assessed zones (Figure 1(a1,a2)). All histological characteristics of these groups corresponded to the norms of the structure and architectonics of the liver tissue of this age group of Wistar rats. In fragments of the liver of rats of the groups 3 and 4, collagen accumulation was observed in the walls of large vessels and the liver parenchyma (Figure 1(a3,a4)). In the samples of rats of group 4, fibrotic liver damage was less pronounced. This was different from the samples of group 4, in which there were signs of the development of parenchymal fibrosis and thickening of the vascular walls with the formation of fibrous septa. At the same time, the increase in fibrotic changes in group 3, estimated using macros from ImageJ, was significantly greater than in samples from group 4 (mean value ± standard deviation 38.76 ± 3.92% vs. 23.52 ± 3.5%, *p* < 0.001, n = 10) (Figure 1c). Additionally, a comparative study of the cellular structure of the tissue showed that both in groups 3 and 4 there were signs of swelling and necrosis of hepatocytes. However, in the samples of group 3, this process was more pronounced and accompanied by fragmentation and/or complete destruction of the nuclei, in contrast to the samples of the group 4, where the nuclei of cell remained completely intact (Figure 1(c3,c4)).

**Figure 2:**
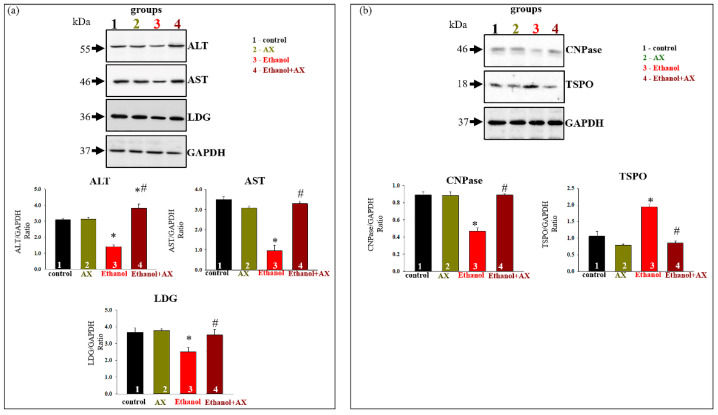
The effect of AX and Ethanol on the content of ALT, AST, LDG, CNPase, and TSPO in rat liver tissue. (**a**)—upper part: immunostaining with ALT, AST, and LDG antibodies; low past: quantification of immunostaining by computer-assisted densitometry presents as a ratio of proteins to GAPDH. GAPDH was used for normalization of proteins. (**b**)—upper part: immunostaining with CNPase and TSPO antibodies; low part: quantification of immunostaining by computer-assisted densitometry presented as a ratio of proteins to GAPDH. The data are presented as the means ± SD of three independent experiments. * *p* < 0.05 significant difference in the protein levels in comparison with the control (group 1). # *p* < 0.05 compared to RLM isolated from ethanol-administrated rats (group 3). The statistical significance of the differences between the pairs of mean values was evaluated using the Student–Newman–Keul test, while ethanol treatment (group 3, red columns on all diagrams) significantly reduced the protein content. Thus, we showed a decrease in ALT levels by 60%, AST by 70%, and LDG by 40% compared with the control group (red columns 3 vs. black columns 1). At the same time, in the liver of rats treated with both ethanol and AX (group 4, scarlet columns in all diagrams), the indicators increased approximately to the control values (scarlet columns 4 compared to black 1). Thus, AX is able to abolish ethanol-induced changes in the liver tissue lysates.

Further, in our experiments, we investigated the alteration in markers of degenerative changes in the liver. Changes in the content of the main indicators of liver dysfunction such as alanine aminotransferase (ALT) and aspartate aminotransferase (AST), as well as lactate dehydrogenase (LDG) in rat liver tissue samples, were tested. The results are shown in Figure 2a. As can be seen from the diagrams in the lower part of the figure, AX treatment did not change the content of AST, ALT, or LDG (dark yellow columns 2 vs 1).

Since the damaging effect of alcohol on the liver is associated with mitochondrial dysfunction, we also checked the content of some of regulatory mitochondrial proteins in the liver tissue. 2′,3′-cyclic nucleotide-3′-phosphodiesterase (CNPase) and translocator protein (TSPO) are involved in the regulation of mitochondrial membrane permeability [29,30,31]. According to our data, they took part in the action of the compensatory system in response to negative effects in mitochondria [32,33,34]. Figure 2b shows that AX administration did not affect the content of TSPO and CNPase, whereas ethanol treatment reduced CNPase content by 40% in tissue lysates compared to control (column 3 vs 1). AX was able to return the CNPase content to control value (column 4 vs 1). Conversely, the content of TSPO increased by two times in RLM from group 3 (ethanol-treated, red column 3 vs 1) and decreased in RLM from group 4 to control value (scarlet columns 4 compared to black 1).

**Figure 3:**
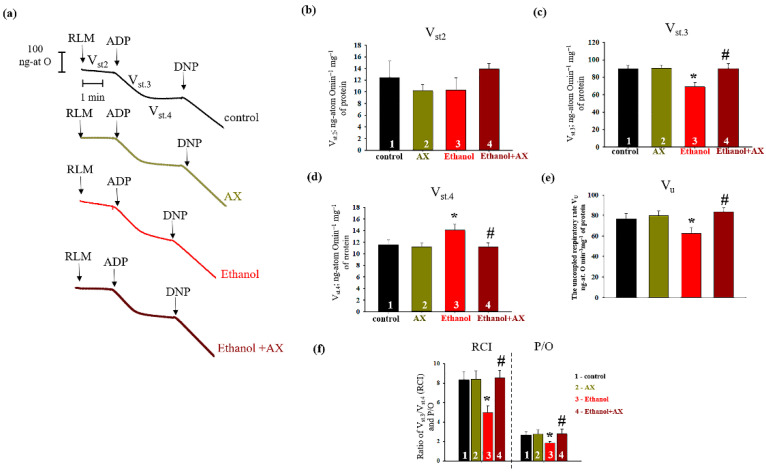
The effects of AX and Ethanol on the change of respiratory activities in RLM. (**a**)—The curves of respiratory activities in state 2 (V_st.2_), 3 (V_st.3_), 4 (V_st.4_), and uncoupled respiration rate (V_u_). Arrows showed the additions to RLM. The concentration of ADP was 200 µM. DNF was 30 µM. (**b**)—quantitative analysis of RLM respiration rate in states 2 (V_st.2_); (**c**)—quantitative analysis of RLM respiration rate in states 3 (V_st.3_); (**d**)—quantitative analysis of RLM respiration rate in states 4 (V_st.4_); (**e**)—quantitative analysis of the uncoupled respiration rate (V_u_); (**f**)—respiratory control index (RCI) values and phosphate/oxygen (P/O) ratio. Oxygen consumption rates (V_st.2_, V_st.3_, V_st.4_, and (V_u_)) were estimated as ng-atom O min^−1^ mg^−1^ protein. The data are presented as the means ± SDs of four independent experiments. * p < 0.05 significant values compared with control (group 1); # *p* < 0.05 significant values compared with RLM isolated from ethanol-administrated rats (group 3).

Since we noticed that AX had an effect on mitochondrial regulatory proteins, we next checked the change in the respiration rates RLM isolated from all experimental groups. The results are presented in Figure 3. Panel 1 (a) shows the curves of mitochondrial respiration, and panel 1 (b–d) indicates the calculations of respiratory rates in different states. We did not notice any changes in the rate of mitochondrial respiration in state 2 (substrate-dependent respiration, V_st.2_) in all experimental groups. Further, no changes occurred in the rate of oxygen consumption in all states in RLM from group 2 (AX-treated) in comparison with control group (columns 2 vs 1 on Panels b, c, and d). However, in RLM from group 3 (ethanol-treated) the rate of oxygen consumption in state 3 decreased by 22%, and in state 4 it increased by 25% compared to control RLM (group 1). In RLM from group 4 (AX + Ethanol-treated), the oxygen consumption rate in states 3 and 4 did not change in comparison with RLM from group 1 (columns 4 vs 1). However, compared to group 3 (AX-treated alone), the rate of oxygen consumption in state 3 accelerated by 24 and in state 4 it slowed down by 25% (columns 4 vs 3) and reached the control values. Figure 3e presents the uncoupled respiration rate (V_u_), a parameter reflecting the magnitude of maximum respiration. The decrease of Vu in the Ethanol RLM compared to the control was 25% (column 3 vs. 1). AX does not cause changes in V_u_ relative to control (column 2 vs 1) but increases its level in ethanol-treated RLM (column 4 vs 3).

Moreover, we calculated two important parameters of the functional state of mitochondria associated with respiratory activity (Figure 3f). Respiratory Control Index (RCI) represents the effectiveness of mitochondria in promoting oxidative phosphorylation and coupling between oxygen consumption and ATP production. The second parameter, P/O, shows the efficiency of oxidative phosphorylation in the mitochondria, defined as the ratio of ATP to absorbed oxygen. The data presented in Figure 3b show that the RCI in RLM from groups 2 and 4 do not differ from the values of the control group 1. However, in group 3, the RCI decreased by 35% compared to the control (column 3 vs 1). The RCI of RLM from group 4 increased by 35% in comparison with the values for group 3 (column 4 vs 3). Similar trends were also observed in the values of P/O. The value of this indicator decreased by 31% in RLM from group 3 compared with group 1, and after AX treatment, P/O increased by approximately 35% in RLM from group 4 relative to that value in RLM from the group 3 (alcohol-treated). This means that AX treatment was able to improve the functional state of mitochondria.

**Figure 4:**
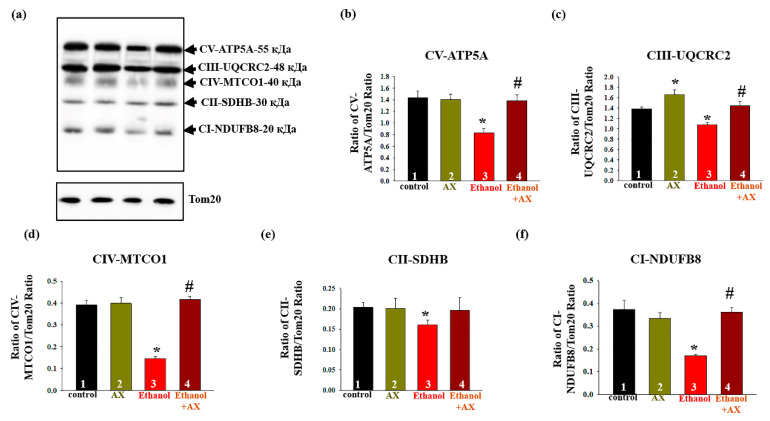
The influence of AX and Ethanol on the change in the levels of the main subunits of the complexes of respiratory chain in RLM. Tom20 was used as a protein load control. (**a**)—immunostaining with an antibody cocktail of OXPHOS; (**b**–**f**)—quantification of immunostaining by computerized densitometry presented as a ratio of subunits to Tom20. The data are presented as mean ± SD of three independent experiments. * *p* < 0.05 indicates a significant difference in the protein level compared to the control (group 1). # *p* < 0.05 compared with the corresponding value in the RLM isolated from ethanol-administrated rats (Group 3). Statistical significance was assessed using the ANOVA type 2 test (Student–Newman–Keuls).

Next, we studied changes in the content of the main subunits of the functional respiratory chain complexes in intact RLM isolated from each group of rats. Figure 4a shows a Western blot stained with OXPHOS antibodies to the basic subunits of the ETC complexes. Tom20 was used as a loading control. Diagrams on Figure 4b–f show quantitative changes in the content of subunits in RLM under the influence of alcohol and upon treatment with AX. It was found that AX does not affect the content of subunits of complexes I, II, IV, and V, but increases the level of cytochrome b-c1 complex subunit 2 of complex III by 20% (Figure 4c, columns 3 vs 1). We found that the effect of ethanol (group 3) significantly decreased the content of subunits of all five complexes by 20–40% (columns 3 vs 1). In the RLM from group 4 (ethanol + AX), the content of subunits of the complexes I, III, IV, and V increased by 60, 20, 60, and 40%, respectively, compared to rats from group 3 that received only ethanol (columns 4 vs 3). At the same time, we did not observe significant changes in the content of the succinate dehydrogenase B of Complex II in RLM from group 4 compared to the RLM from group 3 (Figure 4e).

**Figure 5:**
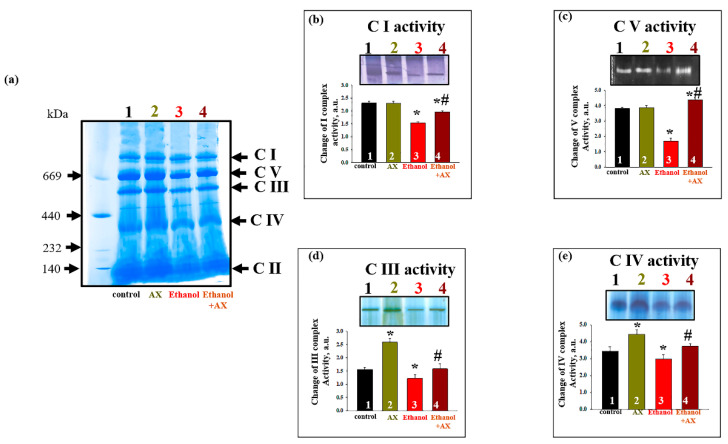
The effect of AX and Ethanol on the activity of respiratory chain complexes and F_o_F_1_-ATP synthase. (**a**)—The native intact RLM isolated from each group was solubilized in the buffer for BNE samples (Materials and Methods Section). Mitochondrial samples were separated by the first dimension BNE, and the gel was stained for the detection of the activity of respiratory chain complexes and F_o_F_1_-ATP synthase (Materials and Methods Section); (**b**)—the change of activity I complex; (**c**)—the change of activity V complex; (**d**)—the change of activity III complex; (**e**)—the change of activity IV complex. The data are presented as mean ± SD of three independent experiments. * *p* < 0.05 indicates a significant difference in the complex activity compared to the control (Group 1). # *p* < 0.05 compared with the corresponding value in the complex activity in RLM from ethanol-administrated rats (Group 3). Statistical significance was assessed using the ANOVA type 2 test (Student–Newman–Keuls).

Since we found that AX could affect the content of subunits of the respiratory chain complexes in the RLM of ethanol-treated rats, we decided to test changes in the activity of complexes under these conditions (Figure 5). The activity of the respiratory chain complexes was measured in gel after BNE, as described in the Materials and Methods section, using appropriate substrates. The gel in Figure 5f represents the distribution of protein complexes after separation of the mitochondrial suspension by BNE (non-denaturing condition). Figure 5b–e show diagrams reflecting changes in the activities of the complexes I, V, III, and IV, respectively. As can be seen in Figure 5b, the activity of the NADH-ubiquinone oxidoreductase, complex I (C I) decreased in mitochondria under the effect of ethanol by 35% (column 3 vs 1). In group 4 (Ethanol + AX), the activity of C I decreased by 20% compared to the control (column 4 vs 1), but it increased by 30% compared to group 3 (only AX treatment, column 4 vs 3). We examined the activity of ATP synthase, complex V (C V) (Figure 5c). No changes in the C V activity of group 2 compared to group 1 were found. Ethanol caused a decrease in C V activity by 60% (column 3 vs 1). In group 4 (ethanol + AX), the C V activity increased by 15% compared with the control (Column 4 vs 1), and almost three times compared with ethanol-treated RLM (Column 4 vs 3). The activity of ubiquinol-cytochrome *c* oxidoreductase (complex III, C III) and cytochrome *c* oxidase (complex IV, C IV) are shown in Figure 5d,e, respectively. Treatment with AX increased the activity of both these complexes by 50% and 30%, respectively (Columns 2 vs 1). In RLM from group 3, the activities of C III and C IV decreased by 20% (columns 3 vs 1). AX treatment abolished the effect of ethanol on C III and C IV activities, thus treatment with AX removed the effect of ethanol, so activity values have been restored to almost 100% (100% was taken as the value of the RLM activity of the control group 1).

**Figure 6:**
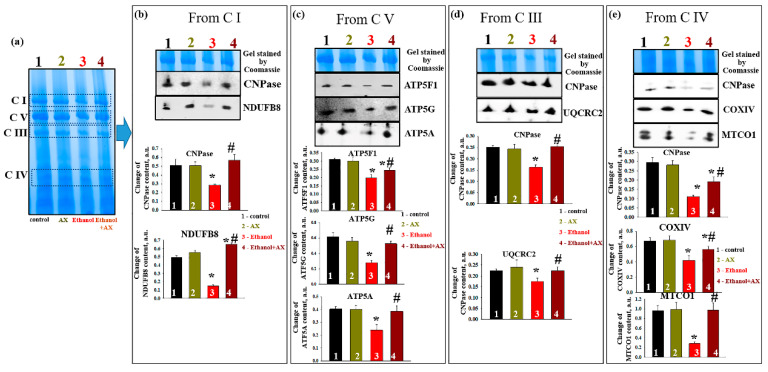
The effect of AX and Ethanol on the change of proteins associated with respiratory chain complexes and F_o_F_1_-ATP synthase. The native RLM were separated to the first dimension by BNE. Bands containing the corresponding complexes were cut out from the gel and applied to the lanes of 12.5% PAAG and separated in the second dimension. The proteins were transferred from the gel to the nitrocellulose membrane. The membrane was stained with the corresponding antibodies. (**a**)—the separation of mitochondrial samples to the first dimension by BNE; (**b**)—the proteins associated with complex I. Upper part: the gel stained with Coomassie, the Western blot stained with CNPase and NDUFB8; low part: the changes of proteins content (absolute units). (**c**)—the proteins associated with complex V. Upper part: the gel stained with Coomassie, the Western blot stained with ATP5F1, ATP5G, and ATP5A; low part: the changes of proteins content (absolute units). (**d**)—the proteins associated with complex III. Upper part: the gel stained with Coomassie, the Western blot stained with CNPase and UQCRC2; low part: the changes of protein content (absolute units). (**e**)—the proteins associated with complex IV. Upper part: the gel stained with Coomassie, the Western blot stained with CNPase, COXIV, and MTCO1. The data are presented as mean ± SD of three independent experiments. * *p* < 0.05 indicates a significant difference in the protein levels compared to the control (Group 1). # *p* < 0.05 compared with the corresponding value in the protein levels in RLM from ethanol-administrated rats (Group 3). Statistical significance was assessed using the ANOVA type 2 test (Student–Newman–Keuls).

In parallel, experiments were carried out to identify changes in the levels of proteins associated with complexes. After separation of mitochondrial proteins into complexes with BNE (the first dimension), strips with complexes were cut out from the gel and subjected to separation in the second dimension by SDS-PAGE [27]. Figure 6a shows an approximate scheme of such a separation. Figure 6b represents changes in the level of proteins associated with C I, namely CNPase and the NADH-dehydrogenase subunit B8 (NDUFB8) of C I. The level of these proteins in C I in the RLM from group 2 did not change compared to group 1, while ethanol decreased the levels of CNPase and NDUFB8 by 50 and 80%, respectively (columns 3 vs 1). Joint treatment of AX and ethanol (group 4) did not affect the level of association of CNPase with C I and led to an increase NDUFB8 content by 30% (columns 4 vs 1). However, compared with the effect of ethanol alone (group 3), the action of AX increased the level of proteins associated with CI in such a way that the level of CNPase increased by 50%, and NDUFB8 increased by six times.

As seen in Figure 6c, AX can also affect the levels of subunits of the ATPase complex (C V) in RLM of ethanol-treated animals (group 4). Under control conditions, AX does not change the levels of subunits *c* (ATP5G), *b* (ATP5F1), and *α* (ATP5A) (columns 2 vs 1). Ethanol intoxication reduced the level of subunits *b* and *α* by 40%, and subunits *c* by 60% (columns 3 vs 1). AX treatment increased levels of subunit *c* by 2-fold, subunit *b* by 30%, and subunit α by 60% relative to RLM in ethanol-dependent rats (group 4 vs group 3).

Next, alteration in the content of CNPase and cytochrome bc1 complex subunit 2 (UQCRC2) in the C III was examined (Figure 6d). Ethanol reduced the levels of the studied proteins by 40% and 20%, respectively (columns 3 vs 1). AX abolished the effect of ethanol, and the content of CNPase increased by 60% and UQCRC2 by 30% (column 4 vs 3). Finally, Figure 6e presents data on changes in protein levels such as CNPase, COXIV, and cytochrome *c* oxidase 1 (MTCO1) in C IV. In the absence of alcohol intoxication, AX did not change the content of CNPase, COXIV, and MTCO1 (columns 2 vs 1). The content of these proteins diminished under the effect of ethanol by 60%, 40%, and 80%, respectively (columns 3 vs 1). The administration of AX increased the level of CNPase in RLM from ethanol-treated rats by 50%, the level of COXIV by 25%, and the level of MTCO1 by 5 times (columns 4 vs 3).

## 4. Discussion

The pathological effect of ethanol on the body is very diverse and affects almost all organs and tissues; however, the liver, which serves as a filter for toxins into which alcohol breaks down, is most affected. Many investigations show that the main cause of degenerative changes in chronic alcoholism is the pathology of mitochondria, namely the dysfunction of pro-apoptotic signaling pathways, oxidative stress, and, as a result, a violation of anti-stress protective mechanisms [32,35,36,37,38,39,40]. However, the mechanism of action of ethanol on mitochondria remains unclear. Therefore, the search for possible targets of potential therapeutic action in the treatment of various ethanol-dependent pathologies continues. It is well known that chronic alcohol consumption is accompanied by oxidative stress [2,3]. Therefore, it is natural to consider antioxidants among possible agents of therapeutic influence. Studies conducted in our laboratory have shown that astaxanthin (AX) is able to have a protective effect on mitochondria in pathologies of the cardiovascular system [20,22]. In addition, the investigations of some researchers demonstrate the suppressive effect of AX on alcohol-induced disorders. Thus, Chang and coauthors showed that AX was able to protect against ethanol-induced gastric injury in rats and inhibited lipid elevation [41]. Other studies suggest that AX may prevent ethanol-induced liver damage by inhibiting oxidative and inflammatory responses [14]. In recent years, AX has been considered a promising mitochondria-targeted agent, and therefore both its antioxidant and other properties in mitochondria are being actively studied [42]. In the present study, we investigated the ability of AX to inhibit the damaging effects of ethanol in rat liver mitochondria.

Four groups of animals were used in the experiments: group 1 (control), group 2 (treated with AX), group 3 (treated with ethanol), and group 4 (treated with ethanol and AX together). A histological analysis of the liver tissue from each group of rats was carried out to identify structural and morphological changes in the liver tissues (Figure 1). In fragments of the liver of groups 3 and 4, fibrous changes were found. However, fibrous liver damage in the samples from group 4 was less pronounced. In addition, in group 3 there were signs of the development of parenchymal fibrosis and thickening of the vascular walls with the formation of fibrous septa. It should also be noted that in the samples from group 3, swelling and necrosis of hepatocytes were accompanied by fragmentation and/or complete destruction of the nuclei, while in the samples from group 4, the cell nuclei remained completely intact. Therefore, AX was able to protect the structure of tissue from degenerative changes caused by ethanol.

Elevated plasma levels of ALT and AST are well-characterized as biomarkers of liver injury [43]. In our case, we analyzed of the levels of AST, ALT, and LDG in liver tissues to assess the achievement of ethanol damage to the liver, as well as to additionally evaluate the effectiveness of AX (Figure 2a). Changes in proteins in tissue lysates confirm the occurrence of ethanol-induced degenerative changes in the liver of the studied animals. AX abolished ethanol effect in the tissue lysates from alcohol-dependent rats. We also showed that AX was able to influence the levels of regulatory proteins of mPTP (Figure 2b). AX increased the level of CNPase in the liver tissue, decreased under the influence of ethanol, and, conversely, reduced the level of TSPO, which was increased under the influence of ethanol. It should be noted that an increased content of TSPO was found in tissues and cells in a whole range of diseases of various etiologies, in particular, cancers of various organs [44,45,46,47,48], aging [49], Alzheimer’s disease [50], and heart diseases [51]. We have recently shown an increase in the level of TSPO in the liver mitochondria of ethanol-dependent rats [32]. We also suggested its participation in the regulation of the protective mechanisms of RLM in chronic alcohol intoxication. Additionally, these mechanisms, in our opinion, include CNPase, which is able to regulate the level of 2′, 3′-cAMP, and 2′,3′-cNADP, and, accordingly, reduce their harmful effects on mitochondria.

We noticed that AX does not affect the parameters of mitochondrial respiration and oxidative phosphorylation in the RLM of control rats (group 2 vs 1). Apparently, this means that under normal conditions, the action of AX does not affect the system of mitochondrial respiration and oxidative phosphorylation, or there are mechanisms that level this action. Decreased values of RCI and the rate of oxidative phosphorylation (V_st.3_) in RLM of ethanol rats compared with controls indicate degenerative changes in the respiratory system of mitochondria. Previously, the negative effect of ethanol on mitochondrial respiration was shown by spectroscopic, polarographic, and microcalorimetric methods [52,53]. These changes lead to disturbances in the systems of electron transport, oxidative phosphorylation, and others, which ultimately leads to mitochondrial dysfunction. However, here we have shown the ability of AX to prevent such disorders in the respiratory system, returning indicators to control values. This confirms the assumption that AX improved the functional state of mitochondria and protected them from the negative effects of ethanol.

Oxidative phosphorylation in mitochondria is mediated by five membrane-bound protein complexes, including NADH ubiquinol oxidoreductase (complex I), succinate ubiquinol oxidoreductase (complex II), ubiquinol cytochrome c oxidoreductase (complex III), cytochrome c oxidase (complex IV), and ATP synthase (complex V). Since the functioning of this system is one of the most important, there is no doubt that defects in the respiratory complexes and ATP synthase affect the function of mitochondria. Here, we have shown that the levels of the basic subunits of the complexes decreased in the RLM during chronic alcohol intoxication, which confirms the disturbances in the functioning of mitochondria (Figure 4). However, AX canceled the effect of ethanol, returning the level of the subunits of the complexes I, III, IV, and V (but not Complex II) to the control values. The flow of electrons through complexes I, III and IV is associated with the movement of protons to create a proton driving force that can drive ATP synthesis or dissipate due to changes in the permeability of the inner membrane [54].

Ethanol is also capable to change the activities of the respiratory chain complexes, and the information on this is rather contradictory. For example, in the 1990s–2000s, studies appeared on the increase in the activity of ATPase under the action of ethanol [55,56]. At the same time, a decrease in the activity and expression of complexes in chronic alcoholism was also shown [39,57,58]. In our experiments, we investigated the changes in the activity of the complexes in the gel after Blue Native Electrophoresis (BNE) (Figure 5). We have shown that the administration of AX increased the activity of complexes III and IV in the RLM compared to control conditions (group 2 vs 1). Under conditions of chronic alcohol intoxication, the activity of all studied complexes decreased (group 3 vs 1). Thus, the chronic effect of alcohol reduces the activity of all complexes of oxidative phosphorylation, which contributes to a decrease in the functioning of the oxidative phosphorylation system and a decrease in the rate of ATP synthesis. This appears to be related to the reduced rate of respiration following the addition of an uncoupler (V_u_) in the mitochondria of alcohol-dependent rats (Figure 3e). We believe that the dysfunction of the complexes in the mitochondria of these rats leads to a decrease in the ability to uncouple. Complexes I, III, and IV are involved in electron transfer and the subsequent creation of a directed proton gradient across the inner mitochondrial membrane. The ATPase uses the free energy of an electrochemical gradient of protons (or sodium ions) generated by the respiratory chain to synthesize ATP. Thus, violation in the complexes leads to destabilization of the proton gradient and disturbances in the production of ATP, which, in turn, causes mitochondrial dysfunction and degeneration. In turn, in the RLM from rats that treated both alcohol and AX, the activity enhanced compared to the RLM from rats that received only alcohol (group 4 vs 3) and returned to control values. Apparently, AX is able to contribute to the maintenance of the normal functioning of the complexes in the RLM in normal conditions and in alcohol intoxication, and even improve it.

There are data in the literature that show the ability of AX to maintain and protect the integrity of mitochondrial ETC and oxidative phosphorylation from oxidative stress [59,60]. However, these studies were carried out on cells, so the nature and significance of this effect is not completely clear. In the present study, we confirmed the protective effect of AX at the organism level. At the same time, revealing the mechanism of action of AX on mitochondrial complexes requires further research. Presumably, it is a consequence of the direct antioxidant action of AX, the induction of antioxidant enzymes, or the remodeling of mitochondrial genes.

A decrease in the activity of the respiratory chain complexes can also occur due to a decrease in the expression and disturbances in the structure of the subunits of these complexes. We additionally studied the expression of proteins associated with the complexes. For this, spots containing complexes were excised from the gel and separated in the second dimension in SDS-PAGE (Figure 6). We have shown that CNPase is associated with complexes I, III, and IV in RLM. At the same time, in chronic alcohol intoxication, the association of CNPase with these complexes decreased, and under the action of AX, it increased to control values (except for C IV, where the effect of AX was less pronounced). We have previously demonstrated a decrease in CNPase expression in mitochondria with aging and ethanol exposure [32,33,34,61]. We also found a decrease in the association of CNPase with complexes during aging and heart failure [22,62]. CNPase is likely able to protect mitochondria from damage by regulating the level of 2′,3′-cAMP. A decrease in the level of CNPase seems to lead to a loss of mitochondrial resistance to stress and damage. The results of this work suggest that CNPase may be one of the targets of AX action in mitochondria. A decrease in the expression of the main subunits of ATPase, namely subunit *α,* subunit *c*, and subunit *b*, was also found under the influence of ethanol. AX abolished this effect, showing its involvement in maintaining cellular energy status and mitochondrial respiratory function. A decrease in the activity and expression of cytochrome *c* oxidase (COXIV) in mitochondria under the influence of ethanol was previously shown [63]. According to our data, under the effect of ethanol, the level of COXIV associated with C IV also decreased, and AX reduced the negative effect of ethanol by increasing the level of COXIV. Finally, it was shown that the association of all key subunits (NDUFB8, ATP5A, UQCRC2, and MTCO1) of the gel-cut complexes after BNE were reduced by ethanol and, accordingly, increased by AX administration. This confirms our results obtained on intact mitochondria using a Western Blot analysis with a Total OXPHOS Rodent WB Antibody Cocktail (Figure 4). All of the above confirms the ability of AX to participate in the mechanisms of the electron transport and energy transfer in mitochondria during alcohol intoxication. For a more detailed understanding of the mechanisms and processes of action of AX, additional studies are required. In particular, we suggest that the non-specific mitochondrial permeability pore (mPTP) is involved in these processes. The study of the effect of AX on the mPTP functioning, as well as a more detailed study of the changes in the ROS production, will be the subject of our next study. However, here we suggest a potential therapeutic benefit of AX against alcohol intoxication.

## 5. Conclusions

The improving effect of AX on the rat liver in alcohol intoxication has been demonstrated. Positive changes included disturbances in the structure and morphology of mitochondria (reducing foci of fibrosis and necrosis of hepatocytes), as well as changes in the levels of the most important biomarkers of liver injury. The accumulation of TSPO is an indicator of the development of a number of diseases, and AX reduced the accumulation of TSPO in the liver of alcohol-dependent rats. AX prevented the reduction of CNPase in liver tissue and the association of CNPase with respiratory chain complexes in mitochondria. In addition, AX improved the functional state of liver mitochondria isolated from alcohol-dependent rats by increasing the rates of respiration and oxidative phosphorylation, as well as the expression of subunits of respiratory chain complexes. Thus, we believe that AX may be involved in the defense system of mitochondria against the pathological effects of alcohol. It is possible that the targets of AX effects are mitochondrial proteins that regulate membrane permeability (CNPase, TSPO) or proteins involved in respiratory chain complexes. All this provides the prerequisites for the use of AX in the treatment of the consequences of diseases associated with the use of ethanol.

## Figures and Tables

**Table 1:** Changes in characteristics after the ethanol treatment of rats. The data are presented as the means ± SD of three independent experiments. * *p* < 0.05 significant difference in the values in comparison with the control (group 1). # *p* < 0.05 compared to RLM isolated from ethanol-administrated rats (group 3). The statistical significance of the differences between the pairs of mean values was evaluated using the Student–Newman–Keul test.

*Parameter*	Groups №			
	1 (Control)	2 (AX)	3 (Ethanol)	4 (AX+Ethanol)
Body weight, g	421.5 ± 8.8	418.7 ± 3.8	390 ± 10.9 *	416.5 ± 3.4 ^#^
Liver weight, g	11.9 ± 0.9	11.5 ± 1.1	13.9 ± 0.3 *	11.3 ± 0.9 ^#^
Ratio of Liver weight/body weight, %	2.8 ± 0.2	2.7 ± 0.3	3.6 ± 0.1 *	2.7 ± 0.2 ^#^
Ethanol content, g/l	-	-	0.9 ± 0.1 *	0.9 ± 0.05 *

## Data Availability

The data presented in this study are contained within this article.
